# SPINNING YOUR WHEELS: AGE DIFFERENCE IN DAILY RUMINATIONS AND COVID-19 STRESS EXPERIENCES

**DOI:** 10.1093/geroni/igad104.2673

**Published:** 2023-12-21

**Authors:** Ann Pearman, Shevaun Neupert, MacKenzie Hughes, Emily Lefebvre

**Affiliations:** MetroHealth Medical System, Cleveland, Ohio, United States; North Carolina State University, Raleigh, North Carolina, United States; Georgia Institute of Technology, Atlanta, Georgia, United States; Vermont Department of Health, Colchester, Vermont, United States

## Abstract

How individuals think about stress affects the impact that stress has on their daily life, however, the nature of this relationship may differ by age and be unique within the context of COVID-19. This study uses a daily diary approach to examine age differences in predictors of daily COVID-19-related stress and appraisals as they unfold. A U.S. sample of 535 adults (white [54%] and Black or African American [46%]) ages 21-79 submitted online diaries for 21 days in 2020. Daily measures included appraisals about how COVID-19 impacted several areas of functioning (e.g. routines, finances), stress due to COVID-19, and amount of rumination about COVID-19. Multilevel modeling showed a significant effect of knowledge on appraisals, where people with more knowledge about the pandemic generally reported that COVID-19 had less impact on their lives. A significant interaction emerged between Age x Daily COVID-19 Stress x Daily COVID-19 Rumination predicting daily appraisals. Whereas the strength of the positive relationship between stress and appraisals remained strong across varying levels of rumination for younger adults, the positive relationship between stress and appraisals was weaker at lower levels of rumination compared to higher levels of rumination for older adults. That is, COVID-19-related stress had less of a perceived impact on older adults’ lives on days they engaged in less rumination about the pandemic. These findings have implications for understanding the role of ruminative thinking on stress and appraisals across the adult lifespan. Different processes may be in play with younger and older adults.

